# Belief in conspiracy theories: Basic principles of an emerging research domain

**DOI:** 10.1002/ejsp.2530

**Published:** 2018-08-24

**Authors:** Jan‐Willem van Prooijen, Karen M. Douglas

**Affiliations:** ^1^ VU Amsterdam Amsterdam The Netherlands; ^2^ The Netherlands Institute for the Study of Crime and Law Enforcement (NSCR) Amsterdam The Netherlands; ^3^ University of Kent Canterbury UK

**Keywords:** conspiracy theories, consequences, universal, emotions, intergroup conflict

## Abstract

In this introduction to the *EJSP* Special Issue on conspiracy theories as a social psychological phenomenon, we describe how this emerging research domain has developed over the past decade and distill four basic principles that characterize belief in conspiracy theories. Specifically, conspiracy theories are *consequential* as they have a real impact on people's health, relationships, and safety; they are *universal* in that belief in them is widespread across times, cultures, and social settings; they are *emotional* given that negative emotions and not rational deliberations cause conspiracy beliefs; and they are *social* as conspiracy beliefs are closely associated with psychological motivations underlying intergroup conflict. We then discuss future research and possible policy interventions in this growing area of enquiry.

Social media and the Internet are filled with conspiracy theories. These theories range from highly implausible in light of logic or scientific knowledge (e.g., chemtrail conspiracy theories; flat‐earth conspiracy theories) to theoretically possible or even plausible (e.g., allegations that secret service agencies routinely violate privacy laws). In fact, conspiracy theories sometimes turn out to be true (e.g., Watergate; incidents of corporate corruption), although the vast majority of conspiracy theories that citizens have believed throughout history have been false (Pipes, 1997). Conspiracy theories are commonly defined as explanatory beliefs about a group of actors that collude in secret to reach malevolent goals (Bale, 2007). What drives belief in such conspiracy theories? While in earlier decades belief in conspiracy theories often was dismissed as pathological (Hofstadter, 1966), accumulating evidence reveals that conspiracy theories are common among surprisingly large numbers of citizens (Oliver & Wood, 2014; Sunstein & Vermeule, 2009). The potential impact and breadth of conspiracy theories was underscored in 2016, when Donald Trump was elected US President despite propagating a range of highly implausible conspiracy theories throughout his campaign. These theories included allegations that climate change is a hoax perpetrated by the Chinese, that Barack Obama was not born in the US, and that vaccines cause autism. The social sciences have increasingly recognized the importance of understanding conspiracy beliefs, and empirical research on this phenomenon has proliferated in the past decade (for overviews, see Douglas, Sutton, & Cichocka, 2017; Van Prooijen, 2018; Van Prooijen & Van Vugt, in press).

The current Special Issue was designed to showcase the study of belief in conspiracy theories as an emerging research domain within social psychology. In putting this issue together, we specifically aimed to capitalize on the momentum that the scientific study of conspiracy theories is currently having, and to give a second generation of conspiracy theory researchers within our field the opportunity to disseminate their novel findings to a professional audience. To introduce this Special Issue, in the present paper we (i) illuminate how the study of conspiracy theories has developed from an unusual object of study to an increasingly expanding research domain over the past few years, and (ii) distill four basic principles that have emerged from past research, in particular that conspiracy beliefs are consequential, universal, emotional, and social. Each of the contributions to this Special Issue considers at least one of these principles. We conclude by proposing a novel research agenda and policy interventions based on these four principles.

## Conspiracy Theories: An Emerging Research Domain

Early studies on conspiracy theories relied mostly on correlational evidence in cross‐sectional designs (e.g., Abalakina‐Paap, Stephan, Craig, & Gregory, 1999; Goertzel, 1994), or studied conspiracy thinking as a function of demographic variables such as political party affiliation (Wright & Arbuthnot, 1974) or ethnicity (Crocker, Luhtanen, Broadnax, & Blaine, 1999). Although scarce and methodologically limited, these early studies provided two key insights that laid the foundations for current research on conspiracy theories. The first key insight is that although conspiracy theories differ widely in content, subjective beliefs in them are rooted in the same underlying psychology. This insight is suggested by findings that the single best predictor of belief in one conspiracy theory is belief in a different conspiracy theory (Goertzel, 1994; see also Lewandowski, Oberauer, & Gignac, 2013; Swami et al., 2011; Sutton & Douglas, 2014). Even beliefs in mutually incompatible conspiracy theories are positively correlated (e.g., Princess Diana was murdered vs. Princess Diana staged her own death; Wood, Douglas, & Sutton, 2012). While many conceptually distinct conspiracy theories exist, the tendency to believe in them appears to be underpinned by broader beliefs that support conspiracy theories in general (e.g., beliefs in cover ups; Wood et al., 2012). Some scholars argue for a *conspiracy mindset* as a relatively stable predisposition to believe in conspiracy theories that varies between persons (Imhoff & Bruder, 2014). Despite the high variability in conspiracy theories—involving topics that range from climate change to chronic illnesses to terrorist attacks—research demonstrates that largely similar and predictable psychological processes drive people's belief in them.

The second key insight is that besides individual differences, belief in conspiracy theories is highly sensitive to social context. For instance, ideological motivations influence political conspiracy beliefs depending on election results (e.g., Democrats believe governmental conspiracy theories particularly if there is a Republican in the White House, and vice versa; Wright & Arbuthnot, 1974; see also Golec de Zavala & Federico, 2018; Uscinski & Parent, 2014; Van Bavel & Pereira, 2018). Moreover, throughout history people have believed conspiracy theories particularly in impactful societal crisis situations, such as during fires, floods, earthquakes, rapid societal change, violence, and wars (McCauley & Jacques, 1979; see also Van Prooijen & Douglas, 2017). Finally, social structures that shape citizens’ feelings of vulnerability increase belief in conspiracy theories, as reflected in findings that feelings of powerlessness predict conspiracy beliefs (Abalakina‐Paap et al., 1999; Imhoff & Bruder, 2014), and that conspiracy beliefs are high particularly among members of stigmatized minority groups (Crocker et al., 1999; Davis, Wetherell, & Henry, 2018; Van Prooijen, Staman, & Krouwel, in press).

Recent research has drawn heavily on these two key insights, by extensively testing how stable individual differences predict a tendency to believe conspiracy theories (Darwin, Neave, & Holmes, 2011; Imhoff & Bruder, 2014; Swami et al., 2011; Van Prooijen, 2017), what causal factors increase belief in conspiracy theories (e.g., Douglas & Sutton, 2011; Van Prooijen & Van Dijk, 2014; Whitson & Galinsky, 2008), what basic cognitive processes are involved when people perceive conspiracies (Douglas, Sutton, Callan, Dawtry, & Harvey, 2016; Van Prooijen, Douglas, & De Inocencio, 2018), and what the consequences are of believing conspiracy theories (Bartlett & Miller, 2010; Douglas & Leite, 2017; Jolley & Douglas, 2014a,b). It is safe to say that the scientific study of conspiracy theories has been emerging over the past decade: Both the body of knowledge on this phenomenon, as well as the number of researchers actively working on it, has expanded rapidly.

One limitation of the current state of affairs in the scientific research domain of conspiracy theories, however, is that the field is lacking a solid theoretical framework that contextualizes previous findings, that enables novel predictions, and that suggests interventions to reduce the prevalence of conspiracy theories in society. Recent review articles have sought to address this limitation by providing a framework that illuminates the motivational basis of conspiracy theories—specifically that conspiracy theories appeal to people for epistemic, existential and social motivational reasons (Douglas et al., 2017), and by developing an evolutionary model—the Adaptive Conspiracism Hypothesis—that specifies how the human tendency to believe conspiracy theories evolved through natural selection (Van Prooijen & Van Vugt, in press). These initiatives notwithstanding, at present the field of conspiracy theories is still in its infancy in terms of theory development. To stimulate further theorizing, we propose four basic principles of belief in conspiracy theories that we distilled from research conducted so far. These four basic principles are supported by many studies and, in conjunction with existing models, may provide an organizing framework for researchers to develop more sophisticated theories and research on this phenomenon.

## Belief in Conspiracy Theories: Four Basic Principles

The four basic principles that we put forward here specify and expand the two key insights discussed earlier—that is, (i) belief in different conspiracy theories is driven by similar psychological processes, and (ii) conspiracy beliefs are highly susceptible to social context. We specifically detail what particular antecedents and consequences are involved in the psychological processes underlying belief in conspiracy theories, and how social context influences people's susceptibility to conspiracy theories. Explicitly, we argue that beliefs in conspiracy theories are *consequential*,* universal*,* emotional*, and *social*. In the following sections, we discuss each of these basic principles in turn.

### Principle 1: Conspiracy Beliefs are Consequential

Even when conspiracy theories are highly unlikely to be true, they have an impact on important life dimensions such as health, interpersonal relationships, and safety. This impact is rooted in the subjective reality of belief. What people believe drives their behavior; but while beliefs sometimes may be flawed or even naive, they may produce behavior that has real consequences (cf., the Thomas Theorem; Thomas & Thomas, 1928). One dimension in particular where conspiracy theories are consequential—and usually detrimental—for perceivers is their health. To illustrate this, imagine for a moment that vaccines actually do cause autism. Who would get themselves and their children vaccinated under those circumstances? But while medical scientists widely agree that vaccines do not cause autism, many citizens firmly believe that the pharmaceutical industry conspires to hide the evidence for such a relationship. This motivates these citizens to deny themselves and their children important vaccines. Empirical research underscores such detrimental health consequences of conspiracy theories for believers: Exposing research participants to anti‐vaccine conspiracy theories lowers their intentions to have a child vaccinated (Jolley & Douglas, 2014b). Moreover, these findings are not specific for health‐related conspiracy theories: More general conspiracy beliefs predict a preference for alternative over regular, evidence‐based medical approaches (Lamberty & Imhoff, 2018).

Furthermore, a surprisingly common conspiracy theory among the African American population is that contraceptives are a form of Black genocide. Belief in this conspiracy theory shapes negative attitudes towards contraceptives and predicts decreased use of contraceptives particularly among men (Thorburn & Bogart, 2005). Relatedly, in South Africa AIDS conspiracy theories are common—stipulating for instance that HIV was deliberately created by humans in the laboratory, and that the pharmaceutical industry promotes the “HIV hypothesis” to sell expensive yet harmful antiretroviral drugs. These conspiracy beliefs are reliably associated with unscientific and dangerous beliefs such as that HIV is harmless, or that condom use causes HIV infections. A study conducted in Cape Town reveals that belief in such AIDS conspiracy theories strongly predicts reduced condom use among both men and women (Grebe & Nattrass, 2012). In fact, one convinced believer of AIDS conspiracy theories was Thabo Mbeki, President of South Africa from 1999 to 2008. Statistical model estimates indicate that in the period between 2000 and 2005, approximately 330,000 South African people died due to governmental decisions not to implement antiretroviral treatment programs (Chigwedere, Seage, Gruskin, Lee, & Essex, 2008).

Belief in conspiracy theories also has implications for people's interpersonal relationships. It has been noted that people who believe conspiracy theories can be subject to stigmatization (Harambam & Aupers, 2015). Consistently, expressing conspiracy theories increases expectations of negative evaluations, and fear of being socially excluded (Lantian, Muller, Nurra, Klein, Berjot, & Pantazi, 2018). Furthermore, evidence suggests that belief in conspiracy theories is associated with problematic interpersonal relationships. Specifically, belief in conspiracy theories is correlated with a range of individual difference variables that reflect impoverished interpersonal functioning, such as interpersonal paranoia (Darwin et al., 2011), narcissism (Cichocka, Marchlewska, & Golec de Zavala, 2016), disagreeableness (Swami et al., 2011), insecure attachment (Green & Douglas, 2018) and Machiavellianism (Douglas & Sutton, 2011). While future research would need to examine the causal effects of conspiracy beliefs on the quality of interpersonal relationships more directly, the findings obtained so far are consistent with the idea that endorsing conspiracy theories is associated with poorer interpersonal functioning.

Conspiracy beliefs also have implications for a range of societal developments. For instance, conspiracy beliefs predict feelings of alienation from politics (Goertzel, 1994), and correspondingly, a manipulation of conspiracy theories decreased participants’ willingness to vote in elections (Jolley & Douglas, 2014a; Study 1). Relatedly, exposure to conspiracy theories decreases public support for important policies. Climate change conspiracy theories—which typically assume that the problem of global warming is a hoax—decrease citizens’ willingness to reduce their carbon footprints (Jolley & Douglas, 2014a; Study 2; see also Douglas & Sutton, 2015), as well as their prosocial behavior more generally (Van der Linden, 2015). Furthermore, conspiracy beliefs are empirically associated with populism (Silva, Vegetti, & Littvay, 2017) and political extremism (Van Prooijen, Krouwel, & Pollet, 2015). Also ‘underground’ extremist movements (e.g., groups of Neo‐Nazis, violent anti‐globalists, religious fundamentalists, and the like) are characterized by excessive conspiracy beliefs. Bartlett and Miller (2010) argued that conspiracy theories causally contribute to the process of radicalization, and the violent tendencies, of such extremist fringe groups.

The above arguments paint a rather bleak picture of the consequences of conspiracy theories and conspiracy beliefs, and indeed, the current state of affairs in this research domain suggests that the majority of consequences are negative. It should be noted, however, that not all consequences are necessarily negative. For instance, conspiracy theories can inspire and justify protest movements (Imhoff & Bruder, 2014; see also Chayinska, Minescu, & Colucci, 2018), and whether that is positive or negative depends on the type of social change that these movements pursue. Furthermore, conspiracy theories can increase governmental transparency (Clarke, 2002), and belief in conspiracy theories is associated with increased support for democratic principles (Swami et al., 2011). Indeed, a fruitful avenue for further research would be to study under what circumstances conspiracy theories are harmful, harmless, or even beneficial. Whether one wishes to focus on the upside or downside of conspiracy theories, one conclusion remains: Conspiracy theories influence citizens, and the society they live in, in significant ways.

### Principle 2: Conspiracy Beliefs are Universal

Conspiracy theories are not restricted to specific times or cultures: Citizens around the world are susceptible to them, from modern to traditional societies (West & Sanders, 2003). Indeed, the tendency to be suspicious of the possibility that others are forming conspiracies against one and one's group may be part of human nature. The Adaptive Conspiracism Hypothesis proposes that while conspiracy theories are not necessarily adaptive in modern environments, they have been adaptive among ancient hunter‐gatherers who faced the problem of frequent intergroup conflict and substantial reproductive loss through coalitional aggression (Van Prooijen & Van Vugt, in press). This model asserts that human beings evolved a conspiracy detection system, that is, a functionally integrated mental system that is activated by specific cues associated with an increased likelihood of hostile coalitions (that is, actual conspiracies), and that produces adaptive outputs to protect ancestral humans from dangerous conspiracies.

While this perspective does not imply that all human beings believe conspiracy theories to an equal extent—individuals, groups, and cultures differ in the extent to which the conspiracy detection system is chronically and situationally activated, as is the case with many other evolved psychological predispositions (Buss, 2009)—it does imply that conspiracy theories are not specific to our modern digital age, or to one particular culture. Empirical evidence supports this view. In their analysis of over a hundred thousand letters sent to major US newspapers between 1890 and 2010, Uscinski and Parent (2014) did not find increased conspiracy theorizing in letters published in the new Millennium; instead, conspiracy theorizing was remarkably stable over a full 120 years. Also Andeweg (2014) found that—contrary to popular belief—satisfaction with politicians did not decrease in an almost 40‐year measurement period (starting in the early 1970s) in multiple EU countries. Instead, citizens’ overall satisfaction with politicians has been low throughout the decades.

Historical sources suggest that substantial numbers of citizens believed conspiracy theories even further back in time. Throughout the centuries, wars were characterized by excessive and mutual conspiracy theories between enemy groups (Pipes, 1997). In Medieval times, conspiracy theories led to major tragedies including the killing of Jews (who were for instance accused of conspiring to poison drinking wells, as a means of explaining disease epidemics) or witch hunts (i.e., young women who were accused of conspiring with the Devil and therefore burnt alive). One can even find conspiracy theories in the writings of the ancient Roman senator and historian Tacitus (Annal XV, 38–44), who described how Roman citizens believed that Nero and his loyal servants deliberately had ignited the great fire of Rome in the year 64 AD (for details, see Brotherton, 2015; Van Prooijen & Douglas, 2017).

Conspiracy theories also appear common to all cultures. While most research conducted thus far on this topic has taken place in Western societies (mostly the US and Western Europe), conspiracy theories are by no means exclusive to these societies. Quantitative research has found evidence for widespread conspiracy beliefs in countries around the world, including Poland (Golec de Zavala & Cichocka, 2012), Ukraine (Chayinska & Minescu, 2018), Malaysia (Swami, 2012), Indonesia (Mashuri & Zaduqisti, 2015), and the Muslim world in the Middle East (Gentzkow & Shapiro, 2004). Ethnographic studies have found substantial conspiracy theorizing in rural Africa (e.g., Namibia; Tanzania) where people endorse a range of conspiracy theories that implicate societal elites, that accuse enemy tribes of witchcraft, or that involve malpractice of the Western world. For instance, many citizens in these regions believe that modern technology is a form of sorcery designed by hostile Western plots to harm or control them (West & Sanders, 2003). Relatedly, anthropologists have observed conspiracy theories among the Yanomamö Amazon Indians in South America, who sometimes blame the mysterious death of a tribe member on sorcery committed by a conspiracy of an enemy village (Chagnon, 1988).

Finally, conspiracy theories emerge across a wide variety of social settings. Conspiracy theories commonly accuse governmental institutions (e.g., politicians in general, or secret service agencies), and entire branches of industry (e.g., the pharmaceutical industry; the oil industry) of malpractice. Furthermore, conspiracy theories often accuse minority groups, such as Muslims or Jews, of hostile plots to plan a revolution (Pipes, 1997). But conspiracy theories also occur in more micro‐level settings. Several studies have revealed that conspiracy theories are common in organizations, where employees suspect their managers of conspiring towards evil goals (e.g., conspiracy theories that managers have a hidden agenda to lay off employees, in order to give themselves a financial bonus; see Douglas & Leite, 2017; Van Prooijen & De Vries, 2016). Although research on the variety of settings in which people believe conspiracy theories is scarce at present, we suspect that conspiracy theories are prevalent also in other domains of social life such as sports (e.g., suspicions that the opposing team bribed the referee, or that supporters of the opposing team plan riots), schools (e.g., suspicions among high‐school students that teachers conspire against them to make exams more difficult), and so on. In any setting characterized by psychological tensions between competing (sub‐)groups, conspiracy theories are likely to occur.

### Principle 3: Conspiracy Beliefs are Emotional

The third principle is partly grounded in a paradox: Conspiracy theories—even blatantly irrational ones—are often supported by a range of elaborate arguments, suggesting that belief in conspiracy theories is based on analytic and deliberative (i.e., System 2) thinking processes. For instance, Moon landing conspiracy theories (assuming that the Moon landings were filmed in a TV studio) often are justified through an extensive analysis of the lack of wind on the moon in conjunction with the apparent movement of the US flag on video recordings. Likewise, many 9/11 conspiracy theories (proposing that these terrorist attacks were an inside job committed by the US government) are based on a range of scientific arguments pertaining to the steel constructions of the former Twin Towers, the maximum temperatures of burning kerosene, and the temperatures at which steel melts. It would therefore be tempting to assume that belief in conspiracy theories is closely associated with an inquisitive mindset that does not take for granted the official readings of impactful events, and that critically analyses evidence in favor of, or against, a conspiracy theory.

Empirical evidence, however, suggests quite the opposite. For example, belief in conspiracy theories is positively associated with intuitive rather than analytic thinking (Swami, Voracek, Stieger, Tran, & Furnham, 2014). Consistently, higher education predicts lower conspiracy beliefs, a finding that is partly mediated by a tendency among the less educated to attribute agency and intentionality where it does not exist (Douglas et al., 2016), and stronger analytic thinking skills among the higher educated (Van Prooijen, 2017). Furthermore, the combination of analytic thinking and the motivation to be rational predicts skepticism of conspiracy theories (Ståhl & Van Prooijen, 2018). It has also been noted that the confirmation bias is central to conspiracy theorizing (Brotherton, 2015), and that conspiracy beliefs are related to the illusion of explanatory depth (Vitriol & Marsh, 2018).

Conspiracy beliefs therefore do not appear to be grounded in controlled, analytic mental processes. Instead, we argue that they are grounded in emotional and intuitive mental (System 1) processes. This insight is based on the argument that aversive emotional experiences increase people's sense‐making motivations (Park, 2010). These sense‐making motivations tend to be sensitive to threats, increasing the likelihood that people attribute suspect events to the covert activities of hostile conspiracies (Hofstadter, 1966). This line of reasoning is consistent with the observation that conspiracy theories gain momentum in the context of anxiety‐provoking societal crisis events such as terrorism, natural disasters, or war (Van Prooijen & Douglas, 2017). The negative emotions that constitute the psychological origins of belief in conspiracy theories include anxiety, uncertainty, or the feeling that one lacks control.

Both correlational and experimental studies extensively support the emotional nature of belief in conspiracy theories. For instance, conspiracy beliefs are correlated with trait anxiety (Grzesiak‐Feldman, 2013), and are predicted by the perception that society is under threat (Jolley, Douglas, & Sutton, 2018), and that society's fundamental values are changing (Federico, Williams, & Vitriol, 2018). Experimental studies have found that inducing a lack of control increases people's belief in organizational conspiracy theories (Whitson & Galinsky, 2008) and political conspiracy theories (Van Prooijen & Acker, 2015). Relatedly, a lack of control leads people to exaggerate the influence that they attribute to their enemies, which is part of many conspiracy theories (Sullivan, Landau, & Rothschild, 2010). Finally, experiencing subjective uncertainty—a phenomenological experience closely associated with lacking control—predicts increased conspiracy beliefs, provided that perceivers consider the implicated authorities as immoral (Van Prooijen & Jostmann, 2013; Whitson, Galinsky, & Kay, 2015).

The sense‐making processes underlying the relationship between emotions and conspiracy beliefs consist of at least two basic and automatic cognitive processes. The first process is pattern perception: People automatically search for meaningful and causal relationships between stimuli. Research indeed finds that perceiving patterns in random stimuli predicts belief in conspiracy theories (Van der Wal, Sutton, Lange, & Braga, 2018; Van Prooijen et al., 2018). The second process is agency detection: People tend to perceive events as caused by intentional agents. The tendency to detect agency in inanimate stimuli empirically predicts belief in conspiracy theories (Douglas et al., 2016; Imhoff & Bruder, 2014). These two basic cognitive processes are reliably triggered by the same emotions that trigger conspiracy beliefs. For instance, lacking control not only increases belief in conspiracy theories but also illusory pattern perception more generally (e.g., seeing images in random noise, or perceiving patterns in random stock market information; Whitson & Galinsky, 2008). In a similar vein, feelings of uncertainty not only increase conspiracy beliefs but also other forms of agency detection, such as people's belief in agentic, moralizing gods (Hogg, Adelman, & Blagg, 2010). While the automatic and epistemic mental processes of pattern perception and agency detection are not emotional per se, aversive emotional experiences do activate these cognitive processes, increasing the likelihood of conspiracy thinking. Taken together, the evidence suggests that belief in conspiracy theories is strongly rooted in negative emotions and automatic processes. The cold, non‐emotional states generally associated with analytic thinking appear to decrease people's belief in conspiracy theories.

### Principle 4: Conspiracy Beliefs are Social

Conspiracy theories are a social phenomenon in that they reflect the basic structure of intergroup conflict. Conceptually, beliefs qualify as conspiracy theories only when they involve assumptions of a hostile and threatening outgroup or coalition (Van Prooijen & Van Vugt, in press). Moreover, these conspiracies typically plan to harm or deceive not just one individual but a wider collective, as is the case with conspiracy theories implicating political organizations, branches of industry, minority groups, managers, and so on. Accordingly, conspiracy beliefs flourish among members of groups who are involved in mutual conflict (Pipes, 1997). Consistently, while belief in conspiracy theories is empirically related to feelings of paranoia (e.g., Darwin et al., 2011), paranoia and conspiracy theories differ in one respect: Paranoia is self‐relevant and necessarily pertains to suspected hostility against a perceiver personally, but instead, conspiracy theories are usually conceived of as intergroup beliefs that assume a powerful or hostile outgroup is conspiring against a perceiver's ingroup (Imhoff & Lamberty, 2018; Van Prooijen & Van Lange, 2014).

Conspiracy beliefs are therefore associated with common motivations that drive intergroup conflict. Two social motivations in particular are relevant for conspiracy thinking. The first motivation is to uphold a strong ingroup identity, which increases perceivers’ sense‐making motivation when they believe their group is under threat by outside forces. That is, people worry about possible conspiracies only when they feel strongly connected with, and hence care about, the prospective victims of these conspiracies. The second social motivation is to protect against a coalition or outgroup suspected to be hostile. This outgroup typically has some threatening quality, such as power (e.g., politicians; managers) or negative stereotypes (e.g., minority groups) which reinforces people's suspicion towards these groups (Douglas et al., 2017; Van Prooijen & Van Lange, 2014). Thus, the combination of a strong ingroup identity and a sense of outgroup threat characterize the social dimension of conspiracy beliefs. These motivations are clearly visible in the political arena, where Republicans often believe conspiracy theories involving Democrats trying to harm Republicans, and Democrats often believe conspiracy theories involving Republicans trying to harm Democrats (Uscinski & Parent, 2014; see also Van Bavel & Pereira, 2018). These effects increase to the extent that people are more polarized in their political ideologies (Van Prooijen et al., 2015).

Empirical research extensively supports these group‐based qualities of conspiracy theories. One source of evidence comes from research on individual differences: Traits that are associated with an increased likelihood of perceiving intergroup conflict also predict increased belief in conspiracy theories. One relevant line of research focused on collective narcissism, that is, exaggerated belief in the greatness of one's ingroup. Feelings of ingroup superiority imply that competing outgroups are considered inferior, which may include the moral inferiority that the main actors in conspiracy theories are assumed to have. Higher scores of collective narcissism indeed predict conspiracy theories that implicate competing outgroups (Cichocka, Marchlewska, Golec De Zavala, & Olechowski, 2016). Furthermore, collective narcissism at the national level predicts how conspiracy beliefs about opposing political parties develop over time during a political election campaign (Golec de Zavala & Federico, 2018).

While findings on collective narcissism primarily emphasize how a strong ingroup identity—in the form of feelings of ingroup superiority—predicts belief in conspiracy theories, other individual difference traits are more directly linked with a structural tendency to perceive outgroups as threatening. Two key individual difference variables commonly connected to stereotyping and intergroup conflict are authoritarianism and social dominance orientation. Several studies have found positive relationships between belief in specific conspiracy theories and these two individual difference variables (Abalakina‐Paap et al., 1999; Imhoff & Bruder, 2014; Swami, 2012). In sum, people who are dispositionally likely to perceive their ingroup as superior or to perceive outgroups as threatening display increased belief in conspiracy theories.

Furthermore, experimental studies support the idea that the two key ingredients of intergroup conflict—a strong ingroup identity and a sense of outgroup threat—jointly stimulate belief in conspiracy theories. For instance, taking the perspective of members of a group increases belief in conspiracy theories, but only after receiving information that the group is under threat (Van Prooijen & Van Dijk, 2014). Likewise, self‐uncertainty predicts increased conspiracy beliefs, but only among people who feel included in a group (Van Prooijen, 2016). These studies suggest that a strong ingroup identity increases conspiracy theories, but only in conjunction with a sense of threat. Experimental studies conducted in Indonesia yielded similar conclusions. People whose Muslim identity was made salient believed conspiracy theories—blaming terrorist attacks in Indonesia on a Western conspiracy—more strongly than people whose Muslim identity was not made salient, but only when the West was described as threatening to Muslims (Mashuri & Zaduqisti, 2015). Finally, basic sense‐making processes predict conspiracy theories only when a hostile outgroup is salient (Marchlewska, Cichocka, & Kossowska, 2018).

Stigmatized minority groups constitute societal examples where these intergroup motivations often are salient. Such groups tend to be highly cohesive, and hence have a strong ingroup identity; at the same time, stigmatized minority groups often suffer from group‐based oppression and discrimination by a more powerful majority group. One would therefore predict that stigmatized minority group members believe conspiracy theories more strongly than majority group members. Research indeed has found substantial conspiracy theorizing among members of minority groups (Goertzel, 1994; Thorburn & Bogart, 2005). Furthermore, stigmatized minority group members believe both identity‐relevant and identity‐irrelevant conspiracy theories more strongly than majority group members (Van Prooijen et al., in press). These effects emerge because minority group members blame the system for realistic problems of their community (i.e., discrimination; see Crocker et al., 1999) and because of a chronic sense of social devaluation (Davis et al., 2018). The social motivations described here provide an explanation why members of marginalized minority groups are particularly likely to believe in conspiracy theories.

Taken together, the findings reviewed in this section underscore the social qualities of conspiracy theories. Even when beliefs in conspiracy theories do not always have prosocial consequences (as illuminated in the section arguing that conspiracy beliefs are consequential), they originate from basic social motivations that characterize intergroup conflict, namely to uphold a strong ingroup identity and to protect against a threatening outgroup.

## Conclusions, Future Research, and Practical Implications

In the present contribution, our aims were to review the literature of the emerging research domain of conspiracy theories, and to distill four basic principles that characterize belief in such theories. These four basic principles follow from a surge of empirical research on this phenomenon that has been conducted in the past decade, and also are reflected in the contributions to this Special Issue. At the same time, more theorizing and research is needed to further develop the psychology of conspiracy theories as a fully‐fledged research field. In the following section, we propose some possibilities for future research based on these four organizing principles.

### Future Research

Focusing first on consequences, whilst it is clear that conspiracy beliefs can have major ramifications for perceivers and their social environment, theorizing on this phenomenon would benefit from more carefully crafted experiments that manipulate conspiracy theories (cf. Douglas & Leite, 2017; Jolley & Douglas, 2014a,b). This would enable researchers to establish the exact psychological processes through which conspiracy theories are consequential. This is important, because only a fine‐grained understanding of these possible consequences, as well as the conditions under which they are strong or weak, will enable practitioners to estimate the risks of particular conspiracy theories and the need to implement preventive interventions. Furthermore, in experimental studies of conspiracy theories, behavioral measurements are also lacking (for an exception, see Van der Linden, 2015). For instance, does exposure to conspiracy theories influence cooperative behavior in economic games? Likewise, do conspiracy theories causally impact antisocial behaviors such as aggression and egoism, but also prosocial behaviors such as helping and altruism? Experimental studies on such questions would complement existing insights on the consequences of conspiracy theories in significant ways.

Next, whilst the available evidence supports the principle that conspiracy beliefs are universal, research needs to more directly and explicitly examine the distal, evolutionary roots of the human tendency to believe conspiracy theories. For instance, while anecdotes exist of conspiracy theories in contemporary hunter‐gatherer societies (Chagnon, 1988), and ethnographic studies suggest that citizens in all cultures investigated so far believe conspiracy theories (West & Sanders, 2003), systematic research on conspiracy theories in traditional societies is currently lacking. The Adaptive Conspiracism Hypothesis asserts that conspiracy theories have been functional in ancient hunter‐gatherer societies to protect against the perils of intergroup conflict (Van Prooijen & Van Vugt, in press). Such lethal intergroup conflict still characterizes many traditional societies: For instance, Walker and Bailey (2013) examined violence in 11 traditional societies in South America and found that an estimated average of 30% of adults in these societies dies through violence, mostly committed by hostile coalitions. Do citizens of violent traditional societies believe conspiracy theories more strongly than citizens of more peaceful traditional societies? And, how functional are conspiracy beliefs in traditional societies to cope with coalitional dangers, as for instance reflected in survival rates and offspring? While these questions appear to be the domain of evolutionary anthropology, they are important to understand why conspiracy theories are such a universal feature of human psychology.

Next, research on the emotional roots of conspiracy belief is restricted to experimentally inducing experiences of threat (e.g., Jolley et al., 2018; Van Prooijen & Acker, 2015; Whitson & Galinsky, 2008) or to measuring threatening or emotional experiences (e.g., Jolley et al., 2018; Federico et al., 2018; Grzesiak‐Feldman, 2013). We would advocate more sophisticated methodologies to study emotions, and particularly recommend a physiological approach to understand the relationship between emotions and belief in conspiracy theories. For instance, the amygdala is commonly associated with threat experiences, and accordingly, bilateral amygdala volume has been found to predict people's tendency to justify the political system that they live in (Nam, Jost, Kaggen, Campbell‐Meiklejohn, & Van Bavel, 2018). As such, brain‐imaging methodology could test the prediction that amygdala volume is associated with conspiracy thinking. Likewise, the dorsolateral prefrontal cortex (DLPFC) is associated with higher‐order cognitive processes such as analytic thinking (e.g., Sanfey, Rilling, Aronson, Nystrom, & Cohen, 2003), and research therefore might examine whether activation of this region predicts belief in, or rather skepticism of, conspiracy theories. Finally, research may examine if belief in conspiracy theories is related with activation of the sympathetic nervous system, or with the release of hormones associated with stress (i.e., cortisol) and intergroup competition (i.e., testosterone).

Regarding the social aspects of conspiracy beliefs, a useful extension would be to focus on actual, real‐life conflict between competing groups. While it has been noted that most wars in which humans have fought have been characterized by excessive conspiracy theorizing on both sides of the conflict, the evidence for this assertion comes mainly from historical sources (Pipes, 1997). As such, empirical research could examine conspiracy theories among existing groups that are involved in intractable, and sometimes violent conflict (e.g., Palestinians vs. Israelis). Predictions that would follow from existing research are that (i) many citizens on both sides of the conflict should have substantial conspiracy beliefs about covert activities of the enemy group, (ii) these conspiracy beliefs should be relatively stronger among members of the (military or politically) “weaker” group in the conflict, and (iii) these effects should be particularly pronounced among citizens with a strong ingroup identity. Furthermore, longitudinal designs to investigate how conspiracy beliefs develop over time are currently scarce (for exceptions, see Golec de Zavala & Federico, 2018; Vitriol & Marsh, 2018). For instance, assessing conspiracy beliefs at multiple time points—ideally, pre‐conflict, during conflict, and post‐conflict—would allow researchers to examine the temporal dynamics of the relationship between conspiracy beliefs and intergroup conflict. Such a longitudinal approach can also establish whether or not conspiracy beliefs cause intergroup conflict or vice versa, and what exact role conspiracy theories play in initiating or prolonging intergroup hostilities (cf. Bartlett & Miller, 2010).

Finally, while our discussion of the social qualities of conspiracy beliefs has mainly focused on intergroup conflict, conspiracy beliefs are also social in the sense that they are highly susceptible to social influence. For instance, online communities selectively spread conspiracy theories that confirm the pre‐existing beliefs of its members (Del Vicario et al., 2016). Furthermore, through cultural transmission conspiracy theories can turn into historical narratives among citizens, which may perpetuate even when the events that triggered the conspiracy theory are no longer salient or threatening (Van Prooijen & Douglas, 2017). An example is the assassination of President John F. Kennedy: Belief in JFK conspiracy theories within the US has increased over the decades. Given the number of people who believe in them (in recent figures still more than 60% of the US adult population; Swift, 2013), they are likely endorsed by many people who were not even born when JFK was assassinated. Yet, much is still unknown about how social influence shapes conspiracy beliefs. For instance, what determines if conspiracy theories spread to a large audience, and what makes them persuasive? What are the characteristics of “successful” conspiracy theories that people still believe years after the events that inspired them? Particularly in the current digital age where information spreads faster than ever before, examining social influence processes in conspiracy beliefs may be a promising avenue for future research.

### Practical Implications

An important task of psychology as a scientific discipline is to inform policy‐makers how to responsibly influence the behavior of citizens based on empirical findings and theoretical insights. That conspiracy theories are consequential and universal underscores a need for interventions: If most of the consequences of conspiracy theories in modern societies are harmful, and if conspiracy theories are widespread in the population, policy‐makers have good reason to take this phenomenon seriously. This does not imply, of course, that our society should abandon efforts to combat actual corruption, or that citizens should uncritically accept any policy proposal of power holders. But, it does imply that many conspiracy theories are irrational yet impactful and harmful, and hence, it is functional to reduce belief in conspiracy theories that are unlikely to be true.

That conspiracy theories are emotional and social offers practical tools for policy‐makers to develop evidence‐based interventions that help to reduce the appeal of conspiracy theories among citizens. First, because belief in conspiracy theories is to some extent rooted in emotions, interventions could instead promote analytic thinking among the public. Research indeed reveals that experimental manipulations designed to stimulate analytic thinking decrease conspiracy beliefs (Swami et al., 2014). Furthermore, providing rational arguments against specific conspiracy theories reduces belief in them (Orosz et al., 2016), and can improve behavioral intentions (Jolley & Douglas, 2017). This suggests that initiatives to refute implausible conspiracy theories (e.g., informing the public what actual experts and witnesses have to say about pseudo‐scientific “9‐11 for truth” conspiracy theories; Dunbar & Reagan, 2011) do make a difference.

The second is to instill feelings of security among the public, and provide them with a sense of hope and empowerment. For instance, if experiencing a lack of control increases conspiracy beliefs, does experiencing empowerment, that is, a *high* sense of control, reduce conspiracy beliefs? Research suggests that this is indeed the case. Van Prooijen and Acker (2015) found reduced conspiracy beliefs after activating a high sense of control as compared to a neutral baseline condition. Likewise, Whitson, Kim, Wang, Menon, and Webster (in press) found similar effects of inducing a promotion focus in participants, and these effects were attributable to increased feelings of control. Future research may expand on the ameliorating effects of more discrete positive emotional experiences on conspiracy beliefs: For instance, are citizens less suspicious of governmental information messages that contain humor? And, are citizens more likely to develop conspiracy theories about pessimistic as opposed to optimistic leaders? For now, evidence suggests that interventions designed to increase analytic thinking and decrease negative emotions may effectively reduce conspiracy beliefs.

While research focusing on the social dimension of conspiracy theories has not yet directly examined how these motivations may be utilized to reduce citizens’ belief in them, an extensive literature exists on how to reduce conflict between groups. For instance, under some circumstances intergroup contact has been found to improve intergroup relations (Allport, 1954). Based on these insights, research may for instance examine whether direct contact between politicians and citizens decreases belief in political conspiracy theories. Specifically, it might be beneficial for public trust if politicians regularly get out of parliament and discuss policy with citizens directly. In a related fashion, emphasizing a superordinate ingroup identity—for instance by engaging in cooperative tasks—may improve intergroup relations (Gaertner, Dovidio, Anastasio, Bachman, & Rust, 1993). This insight might be relevant for the observation that conspiracy beliefs are particularly prevalent among stigmatized minority groups (Crocker et al., 1999; Davis et al., 2018; Van Prooijen et al., in press). Furthermore, among majority group members many conspiracy theories exist in which minority groups are the suspected conspirators (e.g., Pipes, 1997). Efforts to reduce prejudice and discrimination hence are likely to decrease belief in conspiracy theories both among and about minority group members. While preliminary at this point, these considerations suggest that the social qualities of conspiracy theories provide promising avenues for policy interventions.

### Concluding Remarks

The scientific study of belief in conspiracy theories has developed rapidly in the past decade. This development has taken place in the wake of a growing public awareness that conspiracy theories are not exclusive to a few fringe groups or eccentric individuals, but are widespread and have a major impact on society. By organizing the present Special Issue, and by articulating the four basic principles of this research domain in the present contribution, we hope to further stimulate research and inspire other researchers to start working on this important topic. As illuminated in our agenda for future research and policy interventions, there is still much unexplored territory to be discovered in the psychology of conspiracy theories, and scientists and policy‐makers need to collaborate closely to address this phenomenon effectively. We hope that in the end, the empirical contributions to this Special Issue will contribute to decreased conspiracy thinking, and an increased emphasis on logic and reason, among citizens in our society.
